# Challenges of Regulated Cell Death: Implications for Therapy Resistance in Cancer

**DOI:** 10.3390/cells13131083

**Published:** 2024-06-22

**Authors:** Maria D’Amico, Francesca De Amicis

**Affiliations:** 1 Department of Pharmacy, Health and Nutritional Sciences, University of Calabria, 87036 Rende, Italy; 2 Health Center, University of Calabria, 87036 Rende, Italy

**Keywords:** apoptosis, autophagy, ferroptosis, pyroptosis, immunogenic, drug resistance

## Abstract

Regulated cell death, a regulatory form of cell demise, has been extensively studied in multicellular organisms. It plays a pivotal role in maintaining organismal homeostasis under normal and pathological conditions. Although alterations in various regulated cell death modes are hallmark features of tumorigenesis, they can have divergent effects on cancer cells. Consequently, there is a growing interest in targeting these mechanisms using small-molecule compounds for therapeutic purposes, with substantial progress observed across various human cancers. This review focuses on summarizing key signaling pathways associated with apoptotic and autophagy-dependent cell death. Additionally, it explores crucial pathways related to other regulated cell death modes in the context of cancer. The discussion delves into the current understanding of these processes and their implications in cancer treatment, aiming to illuminate novel strategies to combat therapy resistance and enhance overall cancer therapy.

## 1. Introduction

Over the past few decades, extensive experimental evidence provided detailed insights into genetically encoded mechanisms that target and eliminate unnecessary, irreversibly damaged or potentially harmful cells. When mammalian cells encounter irreversible disruptions in their internal or external microenvironment, they may trigger various signal transduction pathways, ultimately resulting in cell death. These regulated cell death (RCD) processes are driven and propagated by complex molecular mechanisms that are highly interconnected. Furthermore, each type of RCD can display a wide range of morphological traits and immunomodulatory properties, spanning from anti-inflammatory and tolerance-inducing effects to promoting inflammation and immunogenic responses [1].

Currently known RCDs mainly include the following: apoptosis, autophagy, pyroptosis, ferroptosis, necroptosis, parthanatos, entosis, cuproptosis, disulfidptosis, lysosome-dependent cell death, alkaliptosis, and immunogenic cell death. These processes have been extensively studied in multicellular organisms, where they play crucial roles in maintaining organismal homeostasis in both normal and disease conditions [2].

Particularly, RCD operates as a natural barrier that protects against cancer development through dedicated molecular machinery. The mechanisms can be targeted through pharmacological or genetic interventions; however, alteration of specific cell death pathways in cancer is often responsible for drug resistance, resulting in therapy failure [3].

The evasion of different cell death mechanisms is considered an indisputable hallmark of cancer [4,5]. For example, cancer cells frequently exhibit overexpression of numerous proteins that are crucial in preventing the initiation of the apoptotic cascade [6]. Most recently, it is emerging that novel forms of RCD are strongly associated with inflammation which, together with a physiological role in the release of cytokines, plays an important role in the modulation of cancer progression and drug resistance [7].

In recent years, numerous studies have elucidated the relationship between RCD mechanisms and the development of cancer across various experimental and clinical models. These investigations expand the theoretical foundation for identifying therapeutic targets, overcoming drug resistance in refractory cancers, and developing prognostic models. However, the pharmacological mechanisms of new drugs are sometimes not well understood, necessitating extensive animal model testing and clinical trials to verify their efficacy [8].

In this concern, programmed death-ligand 1 (PD-L1) translocates to the nucleus of hypoxic cells and upregulates the expression of an important mediator of pyroptosis, Gasdermin C (GSDMC), which is associated with a poor probability of overall survival in breast cancer (BC) [9]. Similarly, the expression of autophagy-related genes like *BECLIN1*, *RAPTOR* and *RICTOR* is associated with the development and progression of colorectal carcinoma (CRC) as well as the emergence of multidrug resistance [10]. Additionally, the expression of lipocalin 2, a siderophore-binding protein that regulates iron homeostasis, is elevated in various tumor types. In colon cancer cell lines, both in vitro and in vivo, this increased expression has been linked to resistance to 5-fluorouracil by inhibiting ferroptosis [11]. In CRCs and hepatocellular carcinoma (HCC), elevated levels of copper transport ATPase (ATP7A) mRNA have also been observed [12]. This suggests that ATP7A could serve as a valuable predictive biomarker for drug resistance in these human cancers. Furthermore, ATP7A plays a pivotal role in the immune tumor microenvironment (TME) and the regulation of immune checkpoints in HCC by modulating the cuproptosis process [13].

However, special attention has been given to observations that, in diverse cases, high-grade cancers with poor prognosis often contain relatively high levels of constitutively dying cells helping the survival, expansion, and evolution of the tumor population [14], suggesting a paradoxal role of apoptosis in cancer biology.

In this review, we will examine the different pathways of RCD and the essential conceptual and mechanistic aspects, along with relative implications for cancer therapy. In the light of diverse functions and regulation in cancer cells we discuss the current knowledge and controversies surrounding the different types of RCD and the possible role for cancer progression and drug resistance. Developing approaches to target different forms of RCD as alternative therapeutics in cancer is imperative and attractive.

## 2. Regulated Cell Death Mechanisms

Traditionally, cell death has been classified based on observable morphological changes; specifically, each type of cell death has been characterized by precise mechanisms for the disposal of dead cells and their fragments. Necrosis, an unordered and passive cellular explosion in response to acute and overwhelming trauma, and apoptosis, widely appreciated as a major mechanism of regulated death, are the two major and diametrically “opposite” modes of cell death. Although initially thought to constitute mutually exclusive cellular states, recent findings reveal cellular contexts that require a balanced interplay between these two modes of cellular demise. Additionally, different types of RCD can exhibit a spectrum of morphological features, ranging from fully necrotic to fully apoptotic. For this reason, classically, RCD was further classified into apoptotic and non-apoptotic cell deaths [15]. In this light, an overview of morphological and biochemical features and biomarkers suggesting several resemblances and differences between RCD mechanisms is provided in Table 1.

More recently, the different types of RCD have been classified according to membrane disruption [16]. Regulated non-necrotic cell death (RNNCD), including apoptosis and autophagy, does not provoke additional inflammatory responses because the cellular components are enclosed within membrane-bound structures, such as apoptotic bodies and autophagosomes, during the cell death process. In contrast, necroptosis, pyroptosis, and ferroptosis, which are morphologically similar to necrosis, are classified as regulated necrotic cell death (RNCD). Due to the membrane disruption associated with RNCDs, these cell death types can trigger inflammatory responses in tissues [17].

More recently, novel signaling pathways governing RCD are being identified. In this review, we focus on the mechanisms of the main RCD subroutines involved in cancer initiation and progression and that are critical for understanding potential implications in cancer therapy resistance.

## 3. Regulated Non-Necrotic Cell Death and Implications in Drug Resistance

### 3.1. Apoptosis

#### 3.1.1. Intrinsic Apoptosis

Intrinsic apoptosis is a type of RCD that can be triggered by various perturbations in the intracellular microenvironment including the withdrawal of growth factors, DNA damage, endoplasmic reticulum (ER) stress, reactive oxygen species (ROS) overload, replication stress, microtubular alterations, or mitotic defects [18]. During intrinsic apoptosis, the cells maintain plasma membrane integrity and metabolic activity as the process progresses. The critical event in intrinsic apoptosis is the irreversibly widespread permeabilization of the outer mitochondrial membrane (OMM), a phenomenon known as mitochondrial outer membrane permeabilization (MOMP) [19] which is crucially mediated by the BCL2 protein family members BAX and BAK.

The pro-apoptotic members of this protein family are activated either transcriptionally or post-translationally in response to specific disruptions in organelles or cellular compartments, thus acting as cellular transducers of stress signaling [20]. MOMP is counteracted by anti-apoptotic members of the BCL2 protein family. The pro-survival proteins are typically inserted into the OMM or the ER membrane through their α9 helix. Their primary function is to exert anti-apoptotic effects by directly binding to pro-apoptotic members of the BCL2 protein family. This interaction between anti-apoptotic and pro-apoptotic proteins helps maintain the integrity of the mitochondria and prevents MOMP, thus promoting cell survival [21,22]. A recent study identified genes whose silencing produced a heterogeneous MOMP phenotype in which some mitochondria within the cell were permeabilized, whereas others remained intact, providing insights into the mechanisms of aberrant apoptosis that are important for developing therapeutic strategies [23].

#### 3.1.2. Extrinsic Apoptosis

Extrinsic apoptosis is initiated by disturbances in the extracellular microenvironment and it is primarily mediated by two types of plasma membrane receptors: death receptors and dependence receptors [24].

Death receptors, such as FAS, cell surface death receptor, and TNF receptor superfamily members, play a crucial role in extrinsic apoptosis through their own activation occurring upon the binding of specific ligands [25]. Next, a dynamic multiprotein complex is assembled at the intracellular tail of the receptors, acting as molecular platforms for regulating the activation and functions of members of the caspase (CASP) family of proteins, such as CASP8 or CASP10, which are both proteolytic enzymes that function as key components of the cell death machinery. It is worth noting that the glycosylation state of some death receptors, such as FAS, can influence the sensitivity of T lymphocytes to extrinsic apoptosis, thereby impacting the termination of inflammatory responses suggesting innovative anticancer therapies [26].

Researchers are beginning to uncover the molecular mechanisms responsible for inducing cell death via dependence receptors when their ligands are absent. These dependence receptors are brought to specific regions on the cell’s outer membrane through a monomeric form interacting with specific CASPs. In the absence of a ligand, complex formation results in caspase activation through a mechanism that sometimes involves caspase cleavage of the receptor itself, thereby releasing proapoptotic peptides [27].

The execution of extrinsic apoptosis involves distinct pathways depending on the type of cells involved. In thymocytes and mature lymphocytes, the activation of CASP8 leads to the proteolytic maturation of executioner CASP3 and CASP7, which is sufficient to induce RCD. This type of apoptosis cannot be inhibited by overexpression of anti-apoptotic BCL2 proteins, the deletion of BAX and BAK1, or the loss of BID [28]. On the other hand, in hepatocytes, pancreatic β cells, and many cancer cells, CASP3 and CASP7 activation is inhibited by the presence of X–linked inhibitor of apoptosis protein (XIAP), an important regulator of apoptosis which effectively blocks the apoptotic cascade, preventing cells from undergoing apoptosis even in the presence of apoptotic signals [29]. In these cells, extrinsic apoptosis requires the proteolytic cleavage of BID by CASP8 [30]. This cleavage generates a truncated form of BID known as tBID, which translocates to the OMM [31].

#### 3.1.3. Apoptosis and Drug Resistance

Extensive literature supports the significant role of defects in apoptotic pathways in the development of cancer, and it suggests that various treatment strategies targeting apoptosis are not only feasible but also applicable to different types of cancer. Despite the frequent dysregulation of apoptosis in tumors, nearly all tumors maintain the core apoptotic regulatory machinery: BCL2 family proteins, cytochrome c (cyt c), CASP, etc. Many of the innovative agents and treatments targeting apoptosis approaches have been integrated into combination therapies with conventional anticancer drugs in numerous clinical trials, aiming to enhance the efficacy of existing treatment modalities [32,33].

However, there are still unresolved questions and concerns surrounding these treatment strategies. One particular concern is whether these approaches could induce resistance in tumors, similar to what has been observed with conventional anticancer drugs [34]. Additionally, there is apprehension regarding the potential for massive cell death of normal cells, which could lead to severe side effects [35].

An interesting facet of cancer cell biology is the relative resistance to apoptosis observed in cancer stem cells (CSCs). These cells, found within a tumor, are particularly adept at initiating tumor formation in transplantation models. In certain cancers, CSCs exhibit higher levels of death receptors (DR), presenting a unique therapeutic target. For instance, the suspected CSC compartment in the human colon cancer cell line SW480 shows elevated levels of DR4; consequently, these CSC SP cells are more responsive to TRAIL. Similarly, in the AT-3 mammary carcinoma cell line, the multipotent, chemoresistant CSC–like population demonstrates higher levels of FAS and DR5 compared to non–CSC–like cells, correlating with increased apoptosis sensitivity induced by the FAS ligand and TRAIL [36]. Ideally, treatment molecules targeting apoptosis would specifically act on a single pathway or protein, providing clinical benefits. A series of high-affinity small organic molecules, known as BH3 mimetics, have been developed to inhibit the interaction between BCL2 and the apoptotic machinery. Venetoclax (formerly ABT-199) is the first of these molecules to receive approval from the US Food and Drug Administration. It is indicated for the treatment of patients with previously treated chronic lymphocytic leukemia (CLL) that features a deletion on the long arm of chromosome 17. Venetoclax effectively inhibits the growth of BCL2–dependent tumors in vivo while sparing human platelets [37].

While drug specificity and selectivity are crucial, it is also important to be broad enough to encompass secondary targets to counteract resistance that may arise due to the presence of these secondary targets. This is the rationale behind the development of “pan-BCL-2” small-molecule inhibitors [38]. Thus, many of the molecules entering clinical trials, such as various inhibitors of the BCL2 family of proteins and pan-inhibitor of apoptosis (IAP) proteins, have multiple targets [39]. This lack of specificity raises concerns about potential off-target effects and unintended consequences.

A good opportunity comes from the therapy-induced senescent cancer cells which play a critical role in impeding cancer progression by inhibiting the growth of oncogenic cells. However, the persistent presence of senescent cells and their ability to evade apoptosis contribute to the adverse effects of chemotherapy and facilitate cancer relapse, leading to therapy resistance [40]. One potential approach to address this challenge is the use of tumor-necrosis-factor-related apoptosis-inducing ligand (TRAIL), a cytokine that can trigger extrinsic apoptosis by binding to DR4 and DR5 [41]. Senescent cells are responsive to TRAIL, and it has been observed that both pro-apoptotic and anti-apoptotic receptors are upregulated in these cells. In particular, a TRAIL variant known as DHER, which selectively binds to DR5, has shown enhanced efficacy in inducing apoptosis in senescent cancer cells compared to wild-type TRAIL [42].

However, targeting apoptosis remains a controversial issue, since a relatively higher level of apoptosis rate is a common feature of the TME and is associated with more aggressive tumors [14,43]. Furthermore, apoptosis may foster genomic instability and niche creation which, in the context of nascent and progressing tumors, could lead to repopulation by tumor cell clones with more aggressive properties. Interestingly, apoptotic cells may attract and polarize macrophages into M2–like states, which can promote cancer development and progression through various pathways. Nevertheless, these so-called tumor-associated macrophages (TAMs) can also participate in antitumor activities, effectively limiting the growth of new and some established tumors [44].

In a fundamental study, authors examined the transcriptomic profile of TAMs in ‘starry-sky’ lymphoma taken from their TME. They found that these TAMs exhibited upregulation of gene clusters related to apoptotic cell clearance, anti-inflammatory responses, survival, proliferation, angiogenesis, and tissue repair/remodeling [45]. Thus, in the context of tumors, a phagocytic clearance of apoptotic cells may impart oncogenic characteristics to the TME. Furthermore, extensive evidence accumulated over the past decade suggests that the TME’s response to apoptosis is modulated at various stages of oncogenesis.

### 3.2. Autophagy-Dependent Cell Death

Autophagy-dependent cell death refers to a specific type of RCD that involves specific molecular machinery or its components [46].

Briefly, the initiation of autophagy is mediated by the Unc-51-like kinase (ULK) complex, which becomes active when mTOR complex 1 (mTORC1) is inhibited or when 5′-AMP-activated protein kinase (AMPK) is activated by stress signals, subsequently activating vacuolar protein sorting 34 (VPS34). The VPS34 complex generates phosphatidylinositol 3-phosphate (PI3P), which serves as a scaffold to recruit PI3P–binding molecules, leading to the formation of an isolated pre-autophagosomal structure, the phagosome. Specifically, PI3P recruits and assembles two ubiquitin-like conjugation systems involved in LC3 lipidation and autophagosome formation. During LC3 lipidation, LC3 is converted to the soluble form LC3I, which then acts as a precursor to LC3II, attached to the phagosome membrane for cargo receptors. The phagosome then extends and closes to form a distinct compartment known as an autophagosome. Autophagosomes are transported to the perinuclear region, where they fuse with nearby lysosomes to form autolysosomes. Within autolysosomes, cargo is degraded by lysosomal hydrolases, and the resulting nutrients are recycled [47].

The process of autophagy is tightly regulated at the transcriptional and post-translational levels and primarily serves as a cytoprotective mechanism in response to stress [48]. However, in several cases, proficient autophagic responses could promote cell survival rather than cell death [49], so that defects in autophagy, either permanent or transient, have been associated with cancer initiation and progression [50]. Very interestingly, autophagy facilitates the engagement of other cell death modalities, such as ferroptosis [51], or through the degradation of the tyrosine phosphatase FAP-1 enhancing FAS–driven extrinsic [52] or intrinsic apoptotic cell death [53,54]. Thus, autophagy could represent a transient event shifting to different molecular processes controlling cell fate.

**Figure 1:**
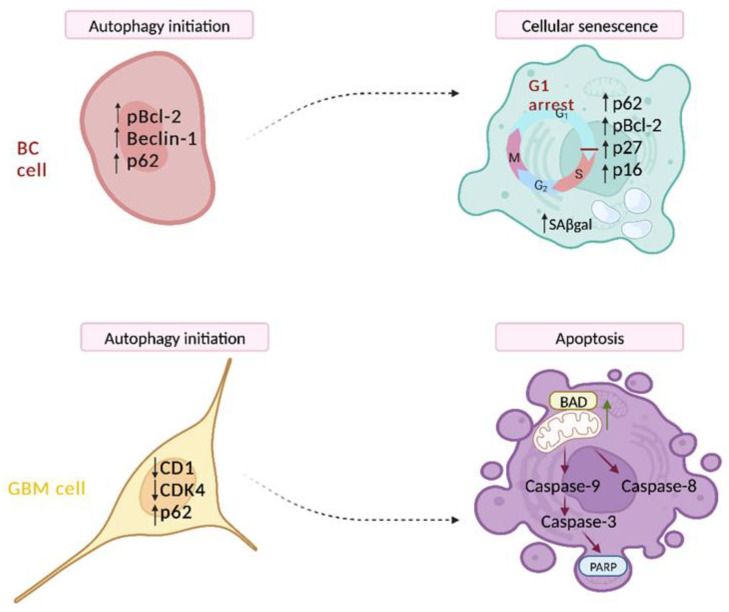
Upper panel (BC cell). A proposed model for the autophagy–senescence switch in BC cells. The increase of Beclin-1 and its release from the inhibitory Bcl-2 interaction due to Bcl-2 phosphorylation enhances the extent of autophagosome assembly. The establishment of an early but incomplete autophagic process causes the induction of p27, which in turn leads to the irreversible inhibition of cell cycle progression and the consequent switch to cellular senescence [55]. Lower panel (GBM cell). A proposed model for the autophagy–apoptosis switch in GBM cells. CDK4 is a key target of γ-secretase inhibitor RO4929097 (GSI) in combination with Resveratrol in GBM cells. The reduction of CDK4/Cyclin D1 levels is responsible for the impaired autophagic flux and the sustained accumulation of p62/SQSTM1. The impaired autophagic flux, providing the block of metabolic intermediates that fuel cell growth and survival, causes the switch towards apoptosis [53]. See the text for details.

In our recent studies in different cancer cells models, we demonstrated a switch from autophagy initiation to senescence or apoptosis. In BC cells (Figure 1 upper panel) we observed that autophagy is an effector of cellular senescence. We discovered that a sustained increase of p-JNK caused BCL2 phopshorylation and the consequent release of Beclin1, which is required for autophagy activation. However, the concomitant accumulation of p62 indicated a deficient autophagic clearance of damaged proteins contributing to cellular senescence, an irreversible cell cycle arrest due to BCL-2 itself, via p27 KIP1 [55]. In a different cancer model such as glioblastoma cells (GBM) (Figure 1 lower panel), the dramatic reduction of an important regulator of lysosomal function such as cyclin-dependent kinase 4 (CDK4) induced an impaired autophagic function, causing the collapse of the system and a following apoptosis. Indeed, the initiator CASP9 and effector CASP3 and the downstream substrate PARP were induced. Thus, a therapeutic combination increasing the accumulation of autophagosomes may have therapeutic value for GBM patients [53].

Autophagy regulation in the context of multi-drug therapy resistance is an expanding area of research that holds a significant position [55,56]. Recent reports highlight the necessity of inducing autophagy for the pulmonary metastasis of HCC cells. In an orthotopic mouse model, stable silencing of autophagic factors Beclin1 and ATG5 in HCC cells hindered the occurrence of pulmonary metastases. Notably, while autophagy inhibition did not impact the migration or invasiveness potential of HCC cells, it did decrease their resistance to cell death and their ability to colonize lung tissue [57].

Key research into oxidative stress and tumor metabolism has revealed a physical convergence of autophagy and aerobic glycolysis within the tumor stroma. Initially, cancer cells release hydrogen peroxide, leading to oxidative stress in cancer-associated fibroblasts. This stress, in turn, triggers autophagy, mitophagy, and aerobic glycolysis. This metabolic interplay reshapes the stroma, allowing for the local generation of recycled and high-energy nutrients like L-lactate, which are utilized to fuel oxidative mitochondrial metabolism in cancer cells. These metabolic models are expected to suggest novel biomarkers and corresponding therapies [58].

Recent studies have revealed the protective role of autophagy in the case of multi-drug therapy resistance, shielding the cancer cells from apoptosis and promoting resistance to chemotherapy. Thus, inhibiting autophagy has shown promise in sensitizing resistant cells to anticancer drugs, making the combination of autophagy inhibitors with cytotoxic drugs [59]. The autophagy inhibitor chloroquine (CQ) and its derivative hydroxychloroquine (HCQ) have gained approval for use in combination with various anticancer drugs to enhance their cytotoxic effects and sensitize refractory cancers [60]. In particular, chronic use of CQ has shown promise in preventing drug resistance, particularly in highly autophagy-addicted tumors such as most aggressive BCs. These findings highlight the potential of combining low doses of CQ with PI3K inhibitors and chemotherapy [56,61].

Equally, a recent study has provided evidence that the downregulation of autophagy-related gene 14 (ATG14) and Forkhead box protein P1 (FOXP1), a well-known transcription factor with oncogenic effects in ovarian cancer cells, can enhance the sensitivity of ovarian cancer cells to cisplatin. The mechanism involves the overexpression of miR-29c-3p, which plays an important role in the malignant progression of tumors [62].

## 4. Regulated Necrotic Cell Death and Implications in Drug Resistance

Tumor cells develop resistance to apoptosis and autophagy by expressing large amounts of specific proteins, which leads to the generation and proliferation of tumors. Because of the ongoing development of targeted therapy, other cell death mechanisms, such as the so-called RNCD, have shown great potential for tumor prevention and treatment [63]. For instance, the continual accumulation of lipid peroxides in the cell membrane ultimately induces ferroptosis, which is an emerging anticancer strategy [64]. However, ferroptosis can trigger inflammation-related immune mechanisms in the TME, leading to tumor growth.

### 4.1. Pyroptosis

Pyroptosis is a form of RNCD widely studied in inflammatory disease models, including cancer [65]. Pyroptosis can be initiated when damage-associated molecular patterns (DAMPs) or pathogen-associated molecular patterns (PAMPs) activate the inflammasomes. Inflammasomes are multimeric protein complexes which trigger the maturation and secretion of pro-inflammatory cytokines modulating immune responses [66]. During pyroptosis, inflammosome activation induces the formation of a huge supramolecular assembly with the adaptor protein ASC, activating CASP1. The activation of CASP1 drives the cleavage of the pro-pyroptotic factor GSDMD [67], generating an N–terminal fragment that oligomerizes to form pores on the host cell membrane and cause the lytic demise of the cell accompanied by the secretion of the proinflammatory mediators, namely high-mobility group protein 1 (HMGB1) IL1-b, IL-18, and IL-1α [68].

Members of the GSDM superfamily are proteolytically activated also by the CASP4/5/11 [69] and CASP3/GSDME signaling pathway, which is a “switch” that can shift the balance between apoptosis and pyroptosis in cancer. When GSDME is highly expressed, CASP3 can cleave GSDME to trigger pyroptosis; otherwise, it triggers apoptosis [70,71].

Emerging evidence indicates a close association between pyroptosis and the development and metastatic progression of various cancers [72]. Particularly, pyroptosis can trigger the crosstalk between innate and adaptive immunity and modulate the TME to induce an immunostimulatory response and promote tumor infiltration and metastasis [73]. Furthermore, the combination of BRAF inhibitors and MEK inhibitors alters the tumor microenvironment by inducing pyroptosis [74].

Interestingly, several cancer cell lines exhibit severe chemoresistance due to the impairment of apoptotic cell death, and the induction of pyroptosis appears promising for cancer treatment [75]. Furthermore, pyroptosis plays a significant role in promoting the efficacy of cancer immunotherapy. For instance, the upregulation of the GSDME protein induces the infiltration of immune cells, such as M1 macrophages and CD4+ and CD8+ T lymphocytes, which increases the sensitivity of cells to anti-PD-1 mAbs [72]. Additionally, studies have revealed that reduced expression levels of the key mediator GSDME of pyroptosis are observed in most tumor cells compared to normal cells, primarily due to mRNA methylation [76]. This reduced expression hampers the activation of pyroptosis in tumor cells. In this concern, during the treatment of malignant tumors, specific chemotherapeutic drugs can be selected based on the expression levels of GSDME. By upregulating GSDME expression in tumor cells, sensitivity to chemotherapeutic drugs can be increased, thereby reducing drug resistance [77].

Very recently, a pyroptosis-related gene (*PRG*) signature (*CASP4*, *CASP9*, *GSDMC*, *IL1A*) was identified and glioma patients were stratified into a low- or high-risk group. Authors concluded that PRG signature is effective in diagnosis and could robustly predict the prognosis of glioma, and its impact on the TME and immune cell infiltrations may provide further guidance for immunotherapy [78].

### 4.2. Ferroptosis 

Ferroptosis is an iron-dependent form of RCD that is triggered by unrestricted lipid peroxidation and subsequent plasma membrane rupture [79]. Iron plays a crucial role as a cofactor for metabolic enzymes, intricately involved in various biological functions, including neurotransmitter transmission, oxygen transportation, mitosis, and energy production. When the regular iron transport mechanisms are disrupted, iron can accumulate within cells. This accumulation can trigger the production of intracellular ROS through the Fenton reaction, a catalytic process that converts ferrous iron and hydrogen peroxide into highly harmful free radicals, contributing to cellular damage. When the intracellular levels of lipid ROS exceed the antioxidant activity of glutathione-dependent peroxidase (GPX4), the leading collapse of cellular redox homeostasis causes ferroptosis. Recently, an antioxidant pathway independent of GPX4 has been found, which relies on coenzyme Q (CoQ) production [80].

Ferroptosis exhibits a specific morphotype characterized by mitochondrial alterations, including shrinkage, electron-dense ultrastructure, reduced or disappeared cristae, and ruptured OMM [81]. Ferroptosis is also potentially linked to the release of DAMPs [82].

The expression of several genes related to iron metabolism, lipid synthesis, and oxidative stress pathways drives ferroptosis [83]. The anti-apoptotic protein BCL2 has been suggested to limit the ferroptosis-mediated physiological demise of neuron progenitors that fail to differentiate. This mechanism is independent of BAX and CASP and can be suppressed by ferroptosis inhibitors. However, the exact role of BCL2 in regulating ferroptosis requires further investigation for confirmation [84]. Compounds such as ferrostatins and liproxstatins inhibit ferroptosis; erastin [85] and RSL3 [86] are ferroptosis inducers. RSL3 directly inactivates GPX4, the main endogenous inhibitor of ferroptosis [87]. GPX4 limits lipid peroxidation by catalyzing the GSH–dependent reduction of lipid hydroperoxides to lipid alcohols [88]. Erastin indirectly affects the catalytic cycle of GPX4 by inhibiting the cystine/glutamate antiporter system, which reduces intracellular cysteine and GSH [89]. Depleting GSH with L-buthionine sulfoximine (BSO), an inhibitor of the glutamate–cysteine ligase complex, can also induce ferroptosis. Additionally, high extracellular glutamate toxicity may involve the activation of ferroptosis through cysteine imbalance [90]. These two main ferroptosis-inducing agents, RSL3 and erastin, have been used in various tumor models [91,92].

Ferroptosis has been found to be associated with resistance to cancer therapy, and inducing ferroptosis has shown potential in reversing drug resistance. Initially, it was associated with RAS mutant cancer cells, but it is now clear that the RAS pathway is not the sole determinant of ferroptosis in tumors [93]. More recently, ferroptosis is emerging as an adaptive mechanism for eliminating malignant cells and represents a novel tumor-suppressing pathway influencing cancer metabolic characteristics. It has been noted that tumor cells experiencing erastin- or RSL3–induced ferroptosis exhibit markedly reduced glycolytic activities. This is indicated by the significant decrease in the expression of three pivotal glycolysis enzymes: hexokinase II, platelet-type phosphofructokinase, and pyruvate kinase M2 (PKM2) [94]. A published study reveals a regulatory pathway that confers resistance to ferroptosis in lung cancer stem-like cells [95]. The authors specifically highlight that the cystine transporter SLC7A11 is overexpressed and can be activated by the stem cell transcription factor SOX2. They found that mutation of the SOX2 binding site in the SLC7A11 promoter reduces SLC7A11 expression and increases sensitivity to ferroptosis in cancer cells. Furthermore, tumors expressing high levels of SOX2 were more resistant to ferroptosis, and there was a positive correlation between SLC7A11 expression and SOX2 in both mouse and human lung cancer tissue. Thus, targeting the oxidation of SOX2 could be a potential therapeutic strategy for cancer treatment.

Notably, the tumor suppressor protein p53, depending on the cellular context, has a dual role in regulating ferroptosis. In certain situations, acetylation-defective p53 mutants can still suppress tumorigenesis through ferroptosis, even when they fail to trigger cell senescence, apoptosis, and cell-cycle arrest [96]. On the other hand, p53 can have an anti-ferroptotic function by boosting antioxidant defenses and limiting ferroptosis induction in CRC [97].

Preclinical and clinical studies have demonstrated that ferroptosis can correlate with cancer therapy resistance. Therapy-resistant high-mesenchymal cell states have been found to depend on GPX4–regulated pathways that protect against ferroptosis [98]. Many chemotherapy drugs can induce ferroptosis, and dysregulation of ferroptosis pathways often leads to chemotherapy resistance [99]. Moreover, evidence suggests that resistance to ferroptosis is a characteristic of metastatic cells, and knocking down GPX4 can reduce the enhanced tumorigenic and metastatic activity of resistant cells [100]. This highlights the relevance of ferroptosis in understanding cancer biology and its potential implications for therapeutic strategies in cancer treatment.

In both intrinsic and acquired resistance, tumor cells have been observed to enhance their defense against oxidative stress by suppressing ferroptosis. This adaptive mechanism allows the cells to survive and become resistant to treatment; thus, reversing cancer resistance may be achieved by inducing ferroptosis [101] and regulating ferroptosis can overcome resistance to conventional chemotherapy, targeted therapy, and immunotherapy [99]. More recently, the role of ferroptosis in T cell immunity and cancer immunotherapy has emerged. Research by Jiang et al. has revealed that suppression of ferroptosis contributes to resistance to antiPD-1/ PD-L1 therapy [102]. These findings suggest that inducing ferroptosis may hold the key to overcoming immunotherapy resistance.

### 4.3. Necroptosis

Necroptosis occurs following the activation of the tumor necrosis receptor (TNFR1) by TNFα [103], but other cellular receptors such as death receptors FAS, Toll-like receptors (TLR4 and TLR3), and cytosolic nucleic acid sensors such as RIG-I and STING, which induce type I interferon (IFN-I) and TNFα production, promote necroptosis in an autocrine feedback loop [104]. Insight into the molecular mechanisms of necroptosis has come primarily through the study of TNFα/TNFR signaling [105] which revealed the essential role of a ‘necrosome’ complex, formed by receptor-interacting protein kinases 1 and 3 (RIPK1, RIPK3). Within the necrosome, activated RIPK3 recruits and phosphorylates mixed lineage kinase domain-like protein (MLKL), which translocates to and permeabilizes the plasma membrane to execute necroptotic cell death [106].

Certain key components of the necroptotic pathway can promote cancer metastasis and progression, potentially contributing to poor outcomes [107]. An elegant recent study strongly suggests the role of necroptosis in cancer progression. Authors demonstrate that Z-DNA–binding protein 1 (ZBP1) mediates tumor necroptosis during tumor development in preclinical cancer models and ZBP1 deletion blocks tumor necroptosis and inhibits metastasis [108].

Furthermore, research conducted by Strilic and colleagues reveals that both human and murine tumor cells induce necroptosis in endothelial cells, facilitating tumor cell extravasation and metastasis. In mouse models, treatment with the RIPK1 inhibitor necrostatin-1 or the endothelial cell-specific deletion of RIPK3 reduced tumor cell-induced endothelial necroptosis, tumor cell extravasation, and metastasis. Conversely, pharmacological inhibition of caspase or endothelial cell-specific loss of caspase-8 promoted these processes. In vivo experiments further illustrated that induced endothelial necroptosis relies on the amyloid precursor protein expressed by tumor cells and its receptor, death receptor 6 (DR6), on endothelial cells, serving as the primary mediators of these effects. These findings suggest that targeting this signaling pathway could be a promising approach for anti-metastatic therapies [109]. Additionally, knocking out essential necroptosis factors like RIP1, RIP3, or MLKL can significantly inhibit cancer cell proliferation in vitro and reduce their ability to form tumors in vivo [110].

Cancer cells evade necroptosis as a survival strategy. In numerous cancer types, a downregulation of key molecules involved in necroptotic signaling pathways, such as RIPK3, has been identified, suggesting that cancer cells may evade necroptosis [111]. Studies have also revealed the tumor-suppressing effects of RIPK3, especially in colorectal cancer, where low RIPK3 expression is linked to reduced disease-free survival and overall survival [112].

There is a growing emphasis on harnessing necroptosis induction as a strategy to combat apoptosis-resistant tumor cells, providing an alternative approach to overcoming resistance to proapoptotic chemotherapeutic agents. Notably, SMAC mimetics, which degrade key anti-cell death mediators like cIAPs, have shown promise when used in conjunction with conventional clinical drugs for the treatment of acute myeloid leukemia (AML). This combination approach triggers necroptosis in apoptosis-resistant AML cells, exploiting the activation of TNFα/RIPK1/RIPK3/MLKL in a synergistic manner [113]. Interestingly, the multitargeting kinase inhibitor sorafenib, utilized in AML treatment, has demonstrated the ability to restrict SMAC mimetic-induced necroptosis in apoptosis-resistant AML cells. This limitation is achieved by inhibiting the phosphorylation of MLKL [114]. However, cancer cells may display resistance to necroptosis inducers. On one hand, cancer cells adapt to the hypoxic conditions of the TME by reprogramming metabolic pathways, notably enhancing anaerobic glycolysis. This metabolic shift may contribute to resistance against RIP1/RIP3–dependent necroptosis, partly due to the scavenging of mitochondrial free radicals by the metabolic product pyruvate. Thus, the hypoxic microenvironment plays a pivotal role in conferring resistance to the induction of necroptosis [115]. Additionally, RIP1 and RIP3 expressions in cancer cells are inclined to be decreased because of genetic mutations or hypoxic induction; thus, pharmaceutical companies may develop new agents that directly target and activate MLKL. For example, Necrosulfonamide can inhibit necroptosis by targeting MLKL directly, in a RIP1- and RIP3–independent manner [116]. Moreover, complementary therapies such as heat therapy and antihypoxia therapy, or the development of MLKL agonists used in combination with necroptotic cancer therapy, hold potential in overcoming resistance to necroptosis [117].

## 5. Other Modalities of Regulated Cell Death and Implications in Drug Resistance

### 5.1. Parthanatos

Parthanatos represents a unique cell-death pathway dependent on the activity of poly (ADP–ribose) polymerase (PARP). Initiated by various stimuli, parthanatos primarily consists of three phases of signal transduction. These phases include the activation of PARP1 and the accumulation of PAR polymer, mitochondrial depolarization, and the translocation of mitochondrial-associated apoptosis-inducing factor (AIF). Possibly occurring at a later stage, CASP activation, although not mandatory, along with large-scale DNA fragmentation and chromatin condensation, ultimately culminates in cell death [118]. This unique sequence of events is of great significance in tumorigenesis, with several key molecules participating in cancer cascades, presenting potential opportunities for developing efficacious treatments. PARP1, PARG, ARH3, and AIF are among these pivotal molecules, which play critical roles in the control of cancer cell growth, progression, invasion, and metastasis [118]. Furthermore, intricate connections exist between parthanatos and other modes of cancer cell death, including apoptosis and autophagy. Thus, a strategic approach involving the simultaneous targeting of multiple cell-death pathways may offer a novel avenue for precise cancer treatment [119].

Due to PARP’s extensive involvement in genomic stability and DNA damage response pathways, the clinical use of PARP1 inhibitors has yielded promising outcomes in patients with certain cancers or specific genetic susceptibilities. Presently, there are more than 250 clinical trials exploring PARP inhibition. Notably, PARP inhibitors such as niraparib, rucaparib, talazoparib, and olaparib have received FDA approval [120].

AIF, the critical downstream effector of PARP1–mediated cell death, plays a crucial role in assembling respiratory complex I and is indispensable for mitochondrial energy production processes. Studies utilizing orthotopically implanted tumors in nude mice indicate that the reduction of AIF levels contributes to the epithelial-to-mesenchymal transition (EMT) process, thereby facilitating cancer cell metastasis. This effect is achieved through oxidative inactivation of the tumor suppressor gene phosphatase and tensin homolog (*PTEN*) [121] and induces the chemoresistance of non-small-cell lung carcinomas [122]. To date, several AIF–targeted therapeutic agents have been utilized in anticancer therapy; for example, the activation of AIF may improve chemotherapeutic treatment in BC [123]. Interestingly, a synthetic cardenolide, which shares structural similarities with the well-known compounds digitoxin and digoxin, triggered parthanatos by overexpressing PARP and PAR in drug-resistant cell lines that overexpress ABC transporters (such as P-glycoprotein and ABCB5). Notably, these effects occurred independently of reactive oxygen species (ROS) [124].

### 5.2. Cuproptosis 

Cuproptosis is a newly discovered type of cell death induced by copper, a crucial trace element that functions as a catalytic cofactor in various biological activities, such as antioxidant defense, mitochondrial respiration, and the synthesis of essential biomolecules [125]. Recent studies have clarified the process of copper-induced RCD [126] through CRISPR/Cas9 screens. Briefly, cuproptosis begins when copper directly binds to lipoylated components of the tricarboxylic acid (TCA) cycle. This interaction causes the aggregation of lipoylated proteins and the degradation of iron–sulfur cluster proteins, leading to proteotoxic stress and subsequent cell death.

Ferredoxin 1 (*FDX1*), a mitochondrial matrix reductase which functions by transferring electrons from NADPH to mitochondrial cytochrome P450, was identified as the key regulatory gene of cuproptosis and the gene most associated with copper ionophore (elesclomol ES) sensitivity [127]. Loss of FDX1 results in a complete loss of protein lipoylation, a significant reduction in cellular respiration, and a diminished response to copper ionophore-induced cell death. FDX1 catalyzes the reduction of ES-Cu2+ to Cu+, facilitating its release into mitochondria. Additionally, FDX1 is an effector of lipoylation, contributing to the accumulation of toxic lipoylated dihydrolipoamide S-acetyltransferase (DLAT). Copper binds to lipoylated DLAT, leading to its toxic accumulation and subsequent cell death [128].

The role of copper in cancer has been extensively studied, revealing significantly higher serum copper ion levels in cancer patients compared to healthy individuals. Moreover, abnormal expression of proteins that regulate copper uptake, distribution, and removal is associated with cancer progression, as evidenced in prostate cancer (PC) [129] and BC [130]. Although copper promotes cancer metastasis by activating metabolic enzymes [131], cuproptosis may contribute to the formation of an antitumor immune environment; however, whether it negatively impacts cancer immunotherapy is still unclear. Cuproptosis is linked to immune cell infiltration, which shapes the TME. A recent study by Wang et al. identified 16 cuproptosis-related long non-coding RNAs (lncRNAs) which can accurately predict the prognosis of lung adenocarcinoma patients. The study revealed that patients classified as high-risk exhibited a higher likelihood of immune escape and a reduced response to cancer immunotherapy [132].

Copper ionophores, including ES, have been evaluated in clinical trials to selectively modify iron and copper levels in cancer cells and to address chemotherapy drug resistance [133]. In various tumor models, including HCC, CRC, Triple Negative BC, and non-small cell lung cancer (NSLC), copper ionophores may enhance the chemosensitizing effect of chemotherapy agents, boost their anti-tumor activity, or counteract chemoresistance [134]. A potent redox modulator, the FDA–approved small molecule Disulfiram (DSF) has been found to selectively enhance the efficacy of radiation and chemotherapy in NSLC, reducing resistance under hypoxic conditions and increasing the antitumor activity of radiation and carboplatin in xenograft tumor models [135]. Although various copper ionophores show a potential in cancer treatments, nevertheless, it is crucial to recognize that these compounds often operate through multiple mechanisms and frequently neglect the role of cuproptosis.

### 5.3. Disulfidptosis

Disulfidptosis, a newly identified form of cell death initiated by disulfide stress, is marked by the disintegration of cytoskeleton proteins and F-actin due to the buildup of intracellular disulfides. This form of cell death occurs specifically in cells with high expression of solute carrier family 7 member 11 (SLC7A11) under glucose starvation conditions, where the lack of repair mechanisms leads to this distinctive cellular demise [136].

SLC7A11 is a cystine/glutamate antiporter, primarily responsible for amino acid transport across the plasma membrane, and it plays a crucial role in cancer cell survival. In 2017, Gan and colleagues discovered that overexpression of SLC7A11 heightened glucose dependence in cancer cells and led to cell death when glucose was deprived [137]. In 2020, they further demonstrated that inhibiting the protein regulator of cytokinesis 1 (PRC1), combined with the induction of activating transcription factor 4 (ATF4), promotes cell death under conditions of glucose starvation [138]. Research indicates that the SLC7A11–mediated reduction of cystine to cysteine relies heavily on reduced nicotinamide adenine dinucleotide phosphate (NADPH), produced via the glucose–pentose phosphate pathway [139]. Under glucose deprivation, NADPH levels in SLC7A11–overexpressing cells are quickly exhausted, causing abnormal accumulation of cystine and other disulfides. This accumulation induces disulfide stress, leading to rapid cell death. Additionally, in SLC7A11–overexpressing cells, proteins showing the greatest increase in disulfide bonds during glucose deprivation were primarily associated with biological processes or pathways related to the actin cytoskeleton and cell adhesion. This results in abnormal disulfide bonding of actin skeleton proteins, leading to subsequent F-actin contraction [140].

A recent study demonstrated the stability of a 23-gene signature in defining disulfidptosis status across various cancer types [141]. Findings indicate significant heterogeneity in disulfidptosis levels both within and among different cancer types. This variability may be influenced by the location and staging of tumors, suggesting that these factors should be taken into account when devising treatment strategies aimed at targeting disulfidptosis [142].

### 5.4. Lysosome-Dependent Cell Death

Lysosome-dependent cell death (LDCD) is a specific cell death pathway initiated by lysosomal membrane permeabilization which results in the release of lysosomal cathepsins and other hydrolases into the cytosol, ultimately leading to cell death [63]. The accumulation of intracellular ROS or lipid peroxides can cause lysosomal rupture, allowing proteolytic enzymes to escape into the cytoplasm and trigger LDCD. This form of cell death is linked to various pathophysiological conditions, including inflammation, tissue remodeling, and cancer.

In cancer, the quantity, composition, and activity of lysosomal hydrolases are often elevated. Lysosomal enzymes such as heparanase and cathepsins promote cancer cell proliferation, angiogenesis, and metastasis, highlighting their potential clinical significance [143]. This characteristic of cancer cells could also provide a basis for therapeutic intervention.

Lysosomes are also known to play a role in resistance to antineoplastic drugs. One plausible explanation for this phenomenon is the sequestration of chemotherapeutic agents within lysosomes [144] which prevents the drugs from binding to their target molecules, thereby reducing their cytotoxic effectiveness. Additionally, it has been suggested that certain lysosome-accumulating agents, such as doxorubicin and tyrosine kinase inhibitors (TKIs), promote the biogenesis of lysosomal compartments, which further increases lysosomal drug sequestration [145]. Another report indicates that the buildup of drugs within lysosomes triggers the release of lysosomal contents through exocytosis [146]. This process aids in transporting drugs out of the cell through a mechanism distinct from the ATP–binding cassette (ABC) superfamily’s multidrug resistance (MDR) efflux transporters [147].

### 5.5. Alkaliptosis

Alkaliptosis is a regulated form of necrosis driven by intracellular alkalization. This process was initially described through the identification of JTC801, a small molecule that targets G protein-coupled receptors (GPCRs), which induces pH–dependent cell death specifically in cancer cells and inhibits tumor growth in mice [148]. JTC801 has been shown to significantly induce cell death in various human PDAC cell lines and primary human PDAC cells by causing intracellular alkalization through the inhibition of carbonic anhydrase 9 (CA9), a pH regulator on the plasma membrane. The induction of alkaliptosis is dependent on the activation of the nuclear factor-κB (NF-κB) pathway, which also mediates cell survival and pro-inflammatory responses in PDAC. The NF-κB-mediated downregulation of CA9 plays a crucial role in JTC801–induced alkaliptosis and tumor suppression in PDAC cells both in vitro and in vivo. Additionally, genetic suppression of the membrane pH regulator solute carrier family 9 member A7 (SLC9A7/NHE7) also leads to alkalinization and tumor suppression in preclinical PDAC models. These findings suggest that manipulating intracellular pH could be a viable strategy for targeting PDAC. Furthermore, inducing alkaliptosis is proposed as a therapeutic approach to overcome venetoclax resistance in AML cells, and targeting NF-κB-dependent alkaliptosis has shown promise in treating venetoclax-resistant AML [149].

### 5.6. Entotic Cell Death

Entosis is a phenomenon characterized by the engulfment of viable cells by non-phagocytic cells of the same or different type. It can occur in both healthy and malignant tissues. After being engulfed, entotic cells have the potential to be eliminated through a controlled form of cell death that occurs within the entotic vacuole, known as the entosome [150].

The initiation of entosis is primarily triggered by the detachment of epithelial cells from the extracellular matrix, resulting in the loss of integrin signaling. Other mechanisms that can induce entosis include deregulated expression of myosins during cell-to-cell contact formation, differences in mechanical properties or responses to metabolic stress in competing cancer cells, and aberrant mitotic rounding during cell division [151]. The process of entotic cell internalization can occur also through cell invasion rather than phagocytosis. It is mediated by the formation of junctions between the engulfing and entotic cells, involving adhesion proteins such as cadherins and catenin alpha 1 [152]. Actomyosin chains accumulate at the cortex of the internalizing cells, driven by the localized activity of RHOA, ROCK1, ROCK2, and DIAPH1, leading to contraction and subsequent engulfment [153]. Actin also plays a role in entosis by promoting pro-invasive cortical plasma membrane blebbing through signaling pathways involving myocardin-related transcription factor (MRTF), serum response factor (SRF), and ezrin (EZR) [154]. Following engulfment, entotic cells are often eliminated through a specific form of cell death that operates independently of BCL2 proteins and CASP.

Entotic cell death has been observed in various types of cancers and is thought to serve as an oncosuppressor mechanism with potential implications for cancer therapy resistance [155]. Although the exact role and implications of entosis in different contexts and diseases require further investigation, it is now considered a potential predictor of cancer prognosis and entosis inducers have been indicated as anticancer drugs [150].

Conversely, a higher number of entotic structures in cancer tissues has been associated with tumor promotion and progression and this may suggest that entosis may actually benefit malignant cells. For example, clinico–histopathological investigations of various cancers, including head and neck squamous cell carcinomas, lung adenocarcinomas and PDAC, have shown a correlation between an increased number of entotic figures and poor outcomes as well as cancer recurrence [150]. Indeed, the internalized cells may be shielded from detrimental environmental factors such as chemotherapy or other unfavorable conditions induced by anticancer drugs, thanks to the protective entotic vacuole formed within the host outer cell [156]. Eventually, the inner entotic cell can be released from the outer cell, potentially leading to chemotherapy failure or cancer recurrence over time. Notably, studies have demonstrated that PC cells utilize entosis as a survival mechanism against treatment with the TKi nintedanib [157]. The phenomenon of entosis has been linked also to the progression of PDAC, particularly in cases involving liver metastasis. A critical player in this context is the neuroepithelial cell transforming gene 1 (*NET1*), which is closely associated with entosis and significantly contributes to the advancement of PDAC. High levels of NET1 are correlated with unfavorable outcomes. Within PDAC cells, the up-regulation of NET1 through entosis gives rise to a highly aggressive subpopulation, potentially propelling the progression of PDAC [158].

### 5.7. Immunogenic Cell Death

For a long time, it was believed that RCD did not trigger an immune response. However, recent research has shown that in specific situations such as cancer, dying cells release their cellular contents, including DAMPs. These DAMPs act as danger signals and have the potential to stimulate the immune system, leading to an inflammatory response.

Specifically, DAMPs attract innate immune cells like neutrophils, macrophages, dendritic cells (DCs), and natural killer cells (NKs) through diverse recognition receptors to promote the maturation and/or activation of these immune cells [159]. This immune activation, however, provides a foundation for the potential triggering of adaptive immune responses in case stressed cells that cannot restore homeostasis and eventually undergo immunogenic cell death (ICD) [160,161]. Immunogenic cell death (ICD) is characterized by a distinct response pattern that involves the initiation of stress at the organelle and cellular levels, ultimately leading to cell death. This process is associated with the exposure, active secretion, or passive release of various damage-associated molecular patterns (DAMPs).

Some known immunostimulatory DAMPs related to ICD include calreticulin (CRT) heat-shock proteins (HSP70 and HSP90), adenosine triphosphate (ATP), HMGB1, type I interferons, and members of the IL-1 cytokine family [162]. Calreticulin is normally found as a soluble protein within the ER, where it serves functions like chaperone activity and the regulation of calcium levels. However, during ICD, CRT is translocated from the ER to the cell surface even before the exposure of phosphatidylserine (PS). This early relocation of CRT to the plasma membrane plays a crucial role as it interacts with CD91 receptors on phagocytes, allowing them to efficiently engulf dying cells. This interaction between CRT and phagocytes, especially dendritic cells (DCs), promotes the uptake of dying cells and subsequently leads to the cross-presentation of tumor antigens, ultimately resulting in the activation of tumor-specific cytotoxic T lymphocyte responses [162].

Extracellular ATP, released from dying cells, plays a crucial role during the induction of robust antitumor immune responses induced by chemotherapy. Notably, when ATP levels are depleted, the immunogenicity of cell death is completely abolished. Inhibiting autophagy, either through genetic or pharmacological means, significantly reduces the secretion of ATP from dying cancer cells, resulting in a dampened immunogenic response. ATP also has immunostimulatory properties by triggering the activation of the NLRP3 inflammasome, leading to the subsequent secretion of IL-1β. This step is essential for the immune system’s response to cell death, particularly in terms of DC–mediated immunogenicity [163].

Another critical feature of ICD is the release of HMGB1, an abundant nuclear nonhistone chromatin-binding protein, from the nucleus into the vicinity of dying cells. HMGB1 exerts potent pro-inflammatory effects by binding to TLR4 on DC, promoting the efficient processing and cross-presentation of tumor antigens derived from dying cells. In a mouse model of tumor vaccination, the use of HMGB1–depleted tumor cells or neutralizing HMGB1 with specific antibodies compromised the mice’s ability to fight tumor development [164]. Moreover, BC patients with a TLR4 loss-of-function allele, which prevents HMGB1 from binding to TLR4, are more susceptible to relapse after radiotherapy or chemotherapy. However, accumulating evidence suggests that the immunogenic properties of HMGB1 depend on its redox state. The reduced form of HMGB1 has been shown to exhibit potent pro-inflammatory activities, in contrast to the inactive oxidized form. In one study, HMGB1 was found to interact with tumor-infiltrating DCs, suppressing nucleic-acid-mediated antitumor activity in mice with established tumors. Nevertheless, this immunosuppressive effect of HMGB1 is primarily observed in established tumors where the tumor environment likely contains high levels of ROS, suggesting that the inactive oxidized form of HMGB1 may be responsible for the observed tolerogenic effects in such scenarios.

During the course of tumor development, neoplastic cells continually interact with and influence the TME, which progressively becomes immunosuppressive. Thus, the immune system plays a pivotal role in both cancer development and treatment. Inducing ICD can help disrupt the immunosuppressive TME, engage innate components, and prime adaptive immunity mediated by T cells, ultimately contributing to long-term control of tumors [165]. In the 4T1 BC model, reducing the levels of the immunoregulatory cytokine Macrophage Migration Inhibitory Factor (MIF), which is frequently overexpressed in various cancers, significantly enhances ICD in vitro, particularly under serum-free conditions. Furthermore, diminishing MIF expression within the primary tumor in vivo triggers a robust anti-tumor immune response characterized by enhanced DC maturation, followed by an increase in IFN–gamma-producing T cells within the tumor [166].

In a recent publication, Musella and colleagues elucidate that inadequate type I interferon (IFN-I) signaling in tumors undergoing immunogenic cell death (ICD) fosters the buildup of cancer stem cells (CSCs) through the activation of the epigenetic regulator lysine demethylase 1B (KDM1B). KDM1B emerges as a compelling target for the advancement of innovative approaches aimed at enhancing anticancer responses driven by ICD [167].

Therapeutic engagement of ICD in cancer may lead to more effective responses by eliciting antitumor immunity. Several classic ICD inducers, including anthracyclines, cardiac glycosides, oxaliplatin, and cyclophosphamide have been extensively used in controlled studies for the inhibition of tumor growth [168]. Interestingly, photodynamic therapy with hypericin has been shown to induce oxidative ER stress, leading to the early exposure of CRT on the cell surface, active secretion of ATP, and passive release of HSP70 and HSP90 during late apoptosis. This approach effectively prevents tumor growth by inducing ICD, even in non-immunized mice [169].

## 6. Conclusions

Significant strides have been made in understanding how oncogenic states influence sensitivity to various RCD pathways. However, the mechanisms allowing cancer cells to persist and proliferate despite these treatments are less clear. Current therapeutic strategies, while inducing resistance and cancer progression, will cause normal cell death in massive numbers. Recognizing the contrasting effects of RCD is crucial in targeted therapy approaches.

**Figure 2:**
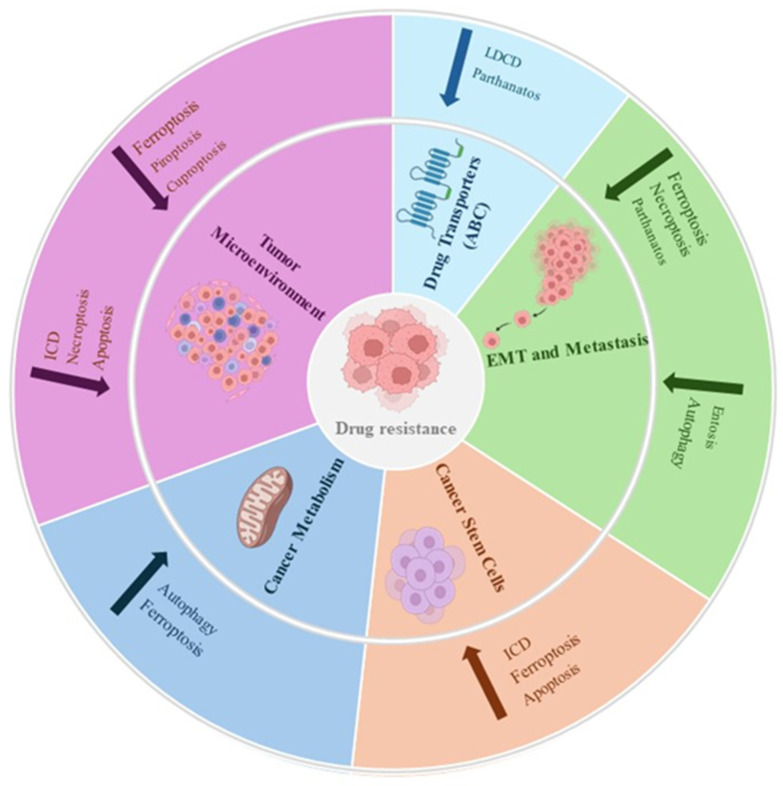
Schematic representation of the crosstalk existing between five hallmarks of cancer drug re-sistance and RCD. Interplay between (1) cancer metabolism and autophagy [58] or ferroptosis [94], (2) stem like phenotype and apoptosis [36], ferroptosis [95] or ICD [167] (3) EMT/metastasis and autophagy [57] or ferroptosis [100], necroptosis [109], parthanatos [121], entosis [158] (4) drug transporters and parthanatos [124] or LDCD [147] (5) tumor microenvironment and apoptosis [44,45] or pyroptosis [73], ferroptosis [102], necroptosis [115], cuproptosis [132], ICD [165] con-verge on drug resistance. See text for details.

Different factors can cause the context-dependent behavior of RCD, and different genes and signaling pathways should be investigated to better understand the effects on cancer (summarized in Figure 2). The proposed concept is that an RCD–sensitive state enables cancer cells to generate mediators capable of modulating intracellular, intercellular, and TME signaling pathways, facilitating cancer growth and progression. Additionally, the abundance of literature underscores the pivotal role of defects in various RCD pathways in carcinogenesis, suggesting novel treatment strategies applicable to various cancer types. Many of these new agents or treatment strategies have also been incorporated into combination therapy involving conventional anticancer drugs in several clinical trials, which may help enhance currently available treatment modalities. Several drugs show a correlation with the effects of regulated cell death in cancer models and significant observations are reported in clinical studies in several cancer types (Table 2 and Table 3).

However, perplexing questions persist. Notably, understanding whether stimulating ferroptosis in diverse cancers can be achieved without triggering pro-carcinogenic inflammation while promoting effective anticancer immune responses is paramount, especially when considering combinations with immune checkpoint inhibitors. This latter point is particularly crucial if novel ferroptosis-inducing regimens are to be combined with immune checkpoint inhibitors, which have become the basis of treatment for most other solid cancers in the gastrointestinal tract.

In summary, investigations into apoptotic and non-apoptotic cell death as strategies to overcome drug resistance in cancer have yielded promising results. These findings open avenues for developing novel therapeutics that leverage different RCD pathways to effectively counteract drug resistance in cancer, spanning traditional chemotherapy, targeted therapy, and immunotherapy.

## Figures and Tables

**Table 1:** Biochemical features and biomarkers for different types of regulated cell death.

	Apoptosis	Pyroptosis	Ferroptosis	Autophagy	Entosis	Necroptosis	Parthanatos	ICD
Pore forming protein	BAX/BAK (intrinsic apoptosis)	GSDMD GSDMB GSDME	Mitochondrial Permeability Transition Pore, ninjurin-1	BID	Not present	Mixed lineage kinase domain-like protein (MLKL)	Ninjurin-1	Not present
Morphologic hallmarks	Shrinkage of the cell, fragmentation into membrane-bound apoptotic bodies	Rapid loss of plasma membrane integrity; cell swelling and rupture	Cell swelling, plasma membrane rupture, smaller mitochondria	Autophagosomes, autolysosome	Cell-in-cell formation	Plasma membrane breakdown, releasing DAMPs	Loss of cell membrane integrity, mitochondrial abnormalities, nuclear shrinkage, and chromatin condensation	DAMPs; global arrest in Transcription and translation
Biochemical hallmarks or Biomarkers	Internucleosomal DNA fragmentation	Phosphatidylserine exposure and caspase activation	Iron accumulation, increased lipid peroxidation	ULK1 complex formation; turnover rate of the ATG8, LC3, p62	Lipidation of LC3 onto the entotic vacuole	Phosphorylation of RIP1, RIP3, and MLKL	DNA fragmentations	Release of type I IFNs, ATP secretion, HMGB1, CALR, and other ER chaperone exposure
Inflammatory nature	no	yes	yes	yes	no	yes	no	yes

**Table 2:** Action of specific drugs in regulated cell death and observations in cancer.

Molecule	Specific Action in Regulated Cell Death	Observations in Cancer Drug Resistance	Type of Cancer Models	Reference
Venetoclax (ABT-199)	Apoptosis inducer	ABT-199 inhibits the de novo pyrimidine synthesis enzyme, leading to overcoming the resistance against hypomethylating agents	Chronic lymphocytic leukemia, follicular lymphoma	[170]
Chloroquine (CQ) Hydroxychloroquine	Autophagy inhibitor	Used in combination with various anticancer drugs to enhance their cytotoxic effects and sensitize refractory cancers.	Central nervous system, lungs, breast, pancreas, leukocytes, skin, and colon/rectum cancers	[60]
Chloroquine (CQ) Hydroxychloroquine	Autophagy inhibitor	Chronic use of CQ has shown to overcome mechanism of drug resistance to PI3K/AKT inhibitors plus paclitaxel	Triple negative breast cancer	[56]
Tyrosine kinase inhibitor Nintedanib	Entosis induction	To inhibit cell proliferation and decrease the growth of xenografts	Prostate cancer	[157]
Multitargeting kinase inhibitor (sorafenib)	Necroptosis inhibitor	To restrict SMAC mimetic-induced necroptosis in apoptosis-resistant cells	Acute myeloid leukemia	[114]
PARP1 inhibitors (olaparib, rucaparib, niraparib and talazoparib)	Parthanatos inhibitors	Anti-tumor efficacy as monotherapy	Tumors expressing either germline or somatic mutations in the *BRCA* genes, advanced/metastatic ovarian cancer, triple negative breast cancer, pancreatic and prostate cancer	[171]

**Table 3:** Approved anticancer drugs targeting regulated cell death mechanisms.

Molecule	Specific Action in Regulated Cell Death	Type of Cancer Models	Reference
5-fluorouracil	Ferroptosis inhibitor	Colon cancer	[11]
BH3-mimetics: Venetoclax (ABT-199)	Apoptosis inducer	Chronic lymphocytic leukemia, follicular lymphoma	[170]
anti-PD-1 (mAbs)	Ferroptosis inducer	Melanoma, lung cancer, metastatic triple negative breast cancer, hepatocellular carcinoma	[102]
Niraparib	Parthanatos inhibitor	Tumors expressing either germline or somatic mutations in the *BRCA* genes, advanced/metastatic ovarian cancer, triple negative breast cancer, pancreatic and prostate cancer	[171]
Rucaparib	Parthanatos inhibitor	Tumors expressing either germline or somatic mutations in the *BRCA* genes, advanced/metastatic ovarian cancer, triple negative breast cancer, pancreatic and prostate cancer	[171]
Talazoparib	Parthanatos inhibitor	Tumors expressing either germline or somatic mutations in the *BRCA* genes, advanced/metastatic ovarian cancer, triple negative breast cancer, pancreatic and prostate cancer	[171]
Olaparib	Parthanatos inhibitor	Tumors expressing either germline or somatic mutations in the *BRCA* genes, advanced/metastatic ovarian cancer, triple negative breast cancer, pancreatic and prostate cancer	[171]
Nintedanib	Entosis inducer	Prostate cancer	[157]
